# Advanced cardiovascular imaging: from patients to populations

**DOI:** 10.1186/1479-5876-10-S2-A6

**Published:** 2012-10-17

**Authors:** David A Bluemke

**Affiliations:** 1 National Institutes of Health Clinical Center, Bethesda, MD 20892, USA

## 

Cardiovascular disease (CVD) is a leading cause of morbidity and mortality in both men and women in developed nations. Gender and population differences in the prevalence, presentation and prognosis of CVD, as well as in the role of traditional risk factors in determining its risk have been increasingly recognized. Thus, multi-ethnic studies are necessary to fully understand the basis for optimal prevention and management of CVD.

Cardiovascular imaging is a well-validated form of non-invasive diagnostic and prognostic testing. Coronary artery calcium (CAC), carotid intima-media thickness (IMT), and elevated left ventricular (LV) mass and geometry as assessed by cardiac magnetic resonance (CMR) offer highly specific phenotype data on the extend of CVD. Due to their high sensitivity, these modalities are increasing used to characterize CVD risk in clinically asymptomatic individuals. Noninvasive imaging of the heart and blood vessels has the potential to replace invasive angiography for the evaluation of ischemic and nonischemic cardiomyopathy. Computed Tomography (CT) angiography allows coronary vessels to be accurately assessed for stenosis. Magnetic resonance imaging (MRI) at 1.5 Tesla and 3 Tesla offers superior evaluation of myocardial structure, function and perfusion, as well as atherosclerotic plaque and tissue composition. In summary, new imaging techniques for the heart and blood vessels offer the potential for advanced tools for patient diagnosis. The application of advanced cardiac CT and MRI to study numbers of patients is underway in epidemiologic studies to help understand risk factor effects and genetic relationships [1,2].
